# Potential Therapeutic Effects of Long-Term Stem Cell Administration: Impact on the Gene Profile and Kidney Function of PKD/Mhm (Cy/+) Rats

**DOI:** 10.3390/jcm11092601

**Published:** 2022-05-05

**Authors:** Daniela Nardozi, Stefania Palumbo, Arif ul Maula Khan, Carsten Sticht, Karen Bieback, Samar Sadeghi, Mark Andreas Kluth, Michael Keese, Norbert Gretz

**Affiliations:** 1 Medical Research Center, Medical Faculty Mannheim, University of Heidelberg, Theodor-Kutzer-Ufer, 68167 Mannheim, Germany; daniela.nardozi@medma.uni-heidelberg.de (D.N.); stefania.palumbo@medma.uni-heidelberg.de (S.P.); arifulmaula.khan@medma.uni-heidelberg.de (A.u.M.K.); carsten.sticht@medma.uni-heidelberg.de (C.S.); 2 Vascular Surgery, University Hospital Mannheim, 68167 Mannheim, Germany; michael.keese@umm.de; 3 Institute of Transfusion Medicine and Immunology, Mannheim Institute of Innate Immunoscience, German Red Cross Blood Service Baden-Württemberg—Hessen, Medical Faculty Mannheim, Heidelberg University, 68167 Mannheim, Germany; karen.bieback@medma.uni-heidelberg.de; 4 RHEACELL GmbH & Co.KG/TICEBA GmbH, 69120 Heidelberg, Germany; samar.sadeghi@ticeba.com (S.S.); andreas.kluth@ticeba.com (M.A.K.)

**Keywords:** cystic kidney disease (CKD), adipose-derived stromal cell (ASC), ABCB5, conditioned media, gene expression profile

## Abstract

Cystic kidney disease (CKD) is a heterogeneous group of genetic disorders and one of the most common causes of end-stage renal disease. Here, we investigate the potential effects of long-term human stem cell treatment on kidney function and the gene expression profile of PKD/Mhm (Cy/+) rats. Human adipose-derived stromal cells (ASC) and human skin-derived ABCB5^+^ stromal cells (2 × 10^6^) were infused intravenously or intraperitoneally monthly, over 6 months. Additionally, ASC and ABCB5^+^-derived conditioned media were administrated intraperitoneally. The gene expression profile results showed a significant reprogramming of metabolism-related pathways along with downregulation of the cAMP, NF-kB and apoptosis pathways. During the experimental period, we measured the principal renal parameters as well as renal function using an innovative non-invasive transcutaneous device. All together, these analyses show a moderate amelioration of renal function in the ABCB5^+^ and ASC-treated groups. Additionally, ABCB5^+^ and ASC-derived conditioned media treatments lead to milder but still promising improvements. Even though further analyses have to be performed, the preliminary results obtained in this study can lay the foundations for a novel therapeutic approach with the application of cell-based therapy in CKD.

## 1. Introduction

Cystic kidney disease (CKD) is a wide group of chronic disorders characterized by the presence of multiple cysts in the kidney and the fourth common cause of end-stage renal disease (ESRD). A genetic mutation is usually the trigger of cyst formation. Autosomal dominant (AD) polycystic kidney disease (PKD), autosomal recessive (AR) PKD and nephronophthisis (NPHP) are the most relevant forms of genetic CKD [1]. ADPKD is a monogenic disorder, with a penetrance of 100% and an incidence of 1:1000 individuals, caused by the mutation of two different genes located on the primary cilia genes of renal tubules: polycystin 1 (PKD1) and polycystin 2 (PKD2) [2,3,4,5]. Several studies investigated the role of these genes, underlining their involvement in kidney development and function [6,7,8,9]. NPHP is a genetically heterogeneous group of CKD, with an autosomal recessive inheritance. There are at least 20 NPHP gene mutations responsible for triggering the disease [10,11,12]. Often, early stages of the disease are asymptomatic; for this reason, the diagnosis is made only when the disease is at a chronic stage or ESRD [13,14]. Nowadays, only a few pharmacological therapies are available for CKD patients, and too often, end-stage patients undergo dialysis and renal transplantation [15,16].

In the early 90s, Gretz et al. characterized an animal model for PKD. A spontaneous mutation resulting in PKD was detected in a Sprague Dawley (SD) colony [17,18,19,20]. In 1997, Bihoreau et al. identified the locus, named *Pkdr1*, of the gene controlling the pathology in this animal model [21]. A few years later, a correlation between a missense mutation on the gene encoding for the SamCystin and the development of cystic disease in the PKD/Mhm rats was found [22]. SamCystin (known as Ank6) is strongly expressed in the kidney tissue and presents multiple ankyrin (ANK) repeats and a sterile α motif (SAM) domain [22,23]. Further studies indicated a correlation between the mutation of Anks6 and the development of NPHP16 [24,25].

The use of mesenchymal stromal cell (MSC)-based therapy is widely used in the treatment of numerous kidney diseases, either acute or chronic due to their anti-inflammatory and antiapoptotic properties, and to their capability to modulate the immune response [26]. Studies have shown how multiple injections of MSC may be a valid therapeutic treatment for acute kidney injury. They demonstrated how repeated human umbilical cord blood mononuclear cell (hUCBMNC) injections could relieve symptoms in rats with acute kidney injuries, reducing apoptosis, oxidative stress and inflammation [27,28].

For the first time, we tested the potential therapeutic effects of two different types of human stem cells and their derived conditioned media on gene expression profiles in the PKD/Mhm (Cy/+) model.

## 2. Materials and Methods

### 2.1. Animal Protocols

Animal experiments were conducted according to the German Animal Protection Law and approved by the local authority (Regierungspräsidium Nordbaden, Karlsruhe Germany in agreement with EU directive 2010/63/EU). We distinguished the heterozygous (Cy/+) animals from non-cystic animals by abdominal palpation. A total of 42 male rats were enrolled and randomly assigned to the base of the received treatment on day 1: 6 rats in the control group and 6 rats in each treatment group (ABCB5^+^-derived CoCM^+^; intraperitoneal (i.p.) ABCB5^+^; intravenous (i.v.) ABCB5^+^; ASC-derived CM; i.p. ASC; i.v. ASC) at the age of 8 weeks. The PKD/Mhm (Cy/+) rats were treated monthly, up to the age of 8 months, with i.p. or i.v. injection of stem cells (2 × 10^6^ cells) or conditioned media injected i.v. (500 μL). GFR measurement and the renal function parameters (plasma and urine samples) were performed every month before every stem cell injection to assess the kidney function.

### 2.2. Characterization of ABCB5^+^ and ASC Cells

The ASC cells were provided by the Institute of Transfusion Medicine of Mannheim. The cells were obtained by lipoaspirates from healthy donors undergoing liposuction, according to Mannheim Ethics Commission II (vote numbers 2010-262 N-MA, 2009-210 N-MA, 49/05 and 48/05) [29,30,31,32]. On the injection day, expanded ASC were trypsinized and suspended in fresh DMEM/10% human serum (blood-type AB) medium at a concentration of 1 × 10^7^ cells/mL, while CM was obtained by collecting the supernatant.

Skin-derived ABCB5^+^ cells were provided by TICEBA GmbH. Skin samples (≥10 cm^2^) were obtained in accordance with the German Medicines Act (“Arzneimittelgesetz”) and the German Act on Organ and Tissue Donation, Removal and Transplantation (“Transplantationsgesetz”) as discard tissues from plastic surgeries (abdominoplasties and mastopexies) from donors aged ≤ 50 years who had given written informed donor consent. The tissues from donors who tested serologically positive for HIV1/2, HBV, HCV, HTLV1/2 or syphilis were discarded. The manufacturing process to isolate ABCB5^+^ cells is fully validated and GMP-certified [33,34]. The supernatant of a co-culture of ABCB5^+^ cells and THP-1-derived macrophages stimulated with IFN-γ and LPS was collected to obtain the ABCB5^+^-derived CoCM^+^ [35].

### 2.3. Tissue Analysis

At the age of 8 months, the animals were deeply anesthetized (Xylazine 5 mg/Kg BW and Ketamine 100 mg/Kg BW) and underwent perfusion (0.9% saline heparin 5 IU/mL pH = 7.0 for 3 min at 280 mbar, 4% PFA for 3 min at 230 mbar). In order to perform the mRNA extraction, the left kidney was collected and snap-frozen in liquid nitrogen prior to perfusion. The right kidney was collected after perfusion and processed for further histological analyses.

#### 2.3.1. RNA Isolation and Sequencing

Total RNA was extracted from whole kidney tissue using the RNeasy mini Kit (QIAGEN) following the manufacturer’s instructions. The Agilent 2100 Bioanalyzer (Agilent (Waldbronn)) was used to determine the RNA purity and integrity. RNA sequencing was performed by BGI Tech Solutions Co. (Hong Kong) using the BGISEQ-500 method. RNA sequencing data were analysed with R Bioconductor software. ENTREZ-based software package TxDb.Hsapiens.UCSC.hg19.knownGene was used, and differential gene expression analysis was performed using a DESeq2 package. A level of significance of α = 0.05 with FDR correction was chosen. A gene set enrichment analysis (GSEA) was performed using KEGG (Kyoto Encyclopaedia of Genes and Genomes) database (GSE161093).

#### 2.3.2. Histology

The kidney tissues were fixed for 24 h in 4% PFA, embedded in paraffin, cut, and stained using haematoxylin and eosin (H&E). The kidney sections were imaged with the slide scanner Axio Scan.Z1 (Zeiss) with a 20× objective. Cyst number and percentage of tissue covered by them were selected and quantified on the whole kidney section using ImageJ (Fiji) software.

#### 2.3.3. Proliferation and Apoptosis Assay

Ki67 immunofluorescence (IF) combined with a Tunel reaction was performed. Briefly, 3 μm kidney sections were pretreated with a citrate buffer, pH 6.0 (20 min, 100 °C), and then incubated in the dark with the Tunel mix (In Situ Cell Death Detection Kit—Fluorescein, ROCHE) for 1h at room temperature (RT). Afterwards, the sections were incubated overnight at 4 °C with the primary rabbit anti-Ki67 antibody (ab15580, Abcam) followed by secondary antibody Alexa Fluor^®^ 647 donkey anti-rabbit (ab150075, Abcam) for 45 min at RT. The samples were counterstained with 4’,6-diamidino-2-phenylindole dihydrochloride (DAPI) for nucleus identification. The slides were imaged with the slide scanner Axio Scan.Z1 (Zeiss) (20× magnification). The whole renal tissue of each scan was evaluated using ImageJ software.

### 2.4. Kidney Function

#### 2.4.1. Biochemistry Analyses

Blood was collected via retro-bulbar vein plexus (orbital sinus) under anaesthesia (5% isoflurane at 3 min/L airflow) in lithium-heparinized tubes using capillaries for blood collection. After centrifugation, plasma was collected in 1.5 mL vials and stored at −20 °C until analysis. The animals were placed into metabolic cages for 16 h in order to collect urine. Afterwards, the urine samples were weighed and centrifuged to remove the precipitate. The urine aliquots were then placed in 2 mL tubes and stored at −20 °C until analysed.

#### 2.4.2. Transcutaneous GFR Measurement

A GFR measurement is considered one of the best indicators of renal function [36]. Therefore, we monitored renal function by evaluating the ABZWCY-HβCD half-life (t1/2) dye. The measurement was performed using a miniaturized transdermal device (MediBeacon GmbH, Mannheim, Germany), which recorded the emitted fluorescence of the ABZWCY-HβCD dye. The procedure was performed as described previously, and the GFRMeasure program (https://github.com/AngeloTorelli/GFRmeasure) was used to calculate the half-life [37,38]. This analysis represents a precise and non-invasive method for measuring kidney function.

### 2.5. Statistical Analysis

The data are presented as mean ± standard deviation (Std Dev). ANOVA and Tukey–Kramer post hoc tests were used to assess the effect of stem cells or conditioned media therapy (JMP^®^ Genomics 7 software). Statistical significance was defined as *p* < 0.05.

## 3. Results

### 3.1. Treatment Effects on Gene Expression

A total of 300 pathways were identified by GSEA. More than 100 pathways were found to be significantly differentially expressed pathways (FDR < 0.05) in almost all of the treated groups (Table 1).

**Figure 1:**
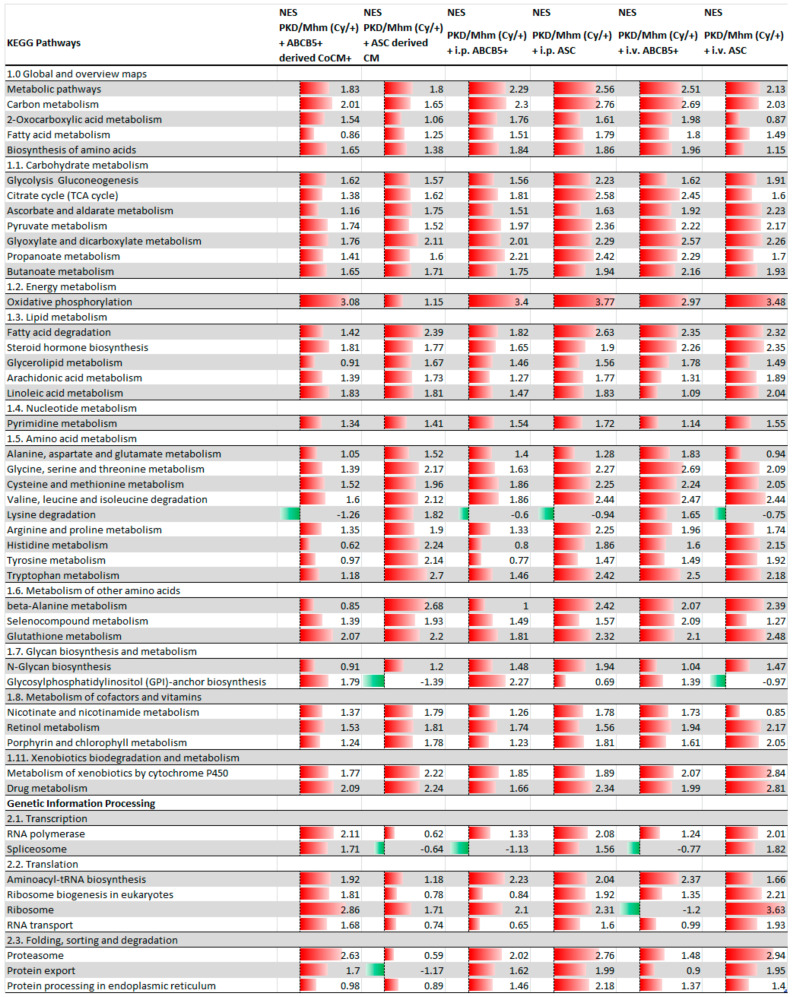
GSEA analysis using KEGG database. Principal upregulated pathways in i.v. ABCB5^+^; i.p. ABCB5^+^; ABCB5^+^−derived CoCM^+^; i.v. ASC groups; i.p. ASC and ASC−derived CM groups. Significantly (*adj. p* < 0.05) differentially expressed pathways (PKD/Mhm (Cy/+) + treatment vs. PKD/Mhm (Cy/+)). Data are sorted by main categories. Normalized enrichment score (NES) is given for each pathway.

**Figure 2:**
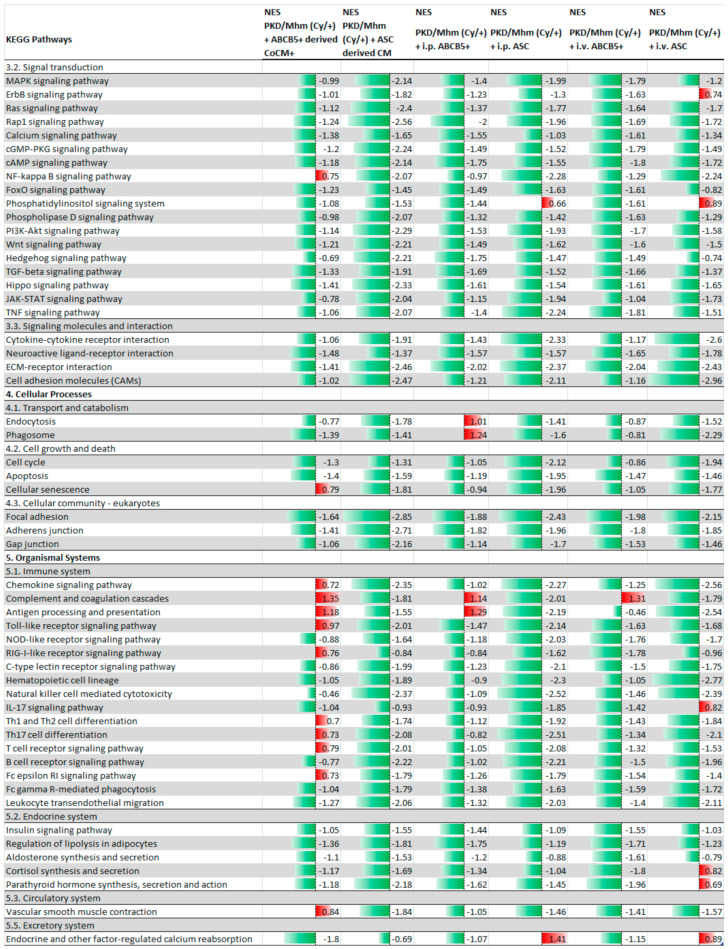
GSEA analysis using KEGG database. Principal downregulated pathways in i.v. ABCB5^+^; i.p. ABCB5^+^; ABCB5^+^—derived CoCM^+^; i.v. ASC groups; i.p. ASC and ASC—derived CM groups. Significantly (*adj. p* < 0.05) differentially expressed pathways (PKD/Mhm (Cy/+) + treatment vs. PKD/Mhm (Cy/+)). Data are sorted by main categories. NES is given for each pathway.

The administration of ASC and ABCB5^+^ cells and their derived conditioned media showed an upregulation of the principal metabolism-related pathways, indicating a shift in the metabolism pathways from glycolysis to gluconeogenesis. In fact, upregulation of the citrate cycle and oxidative phosphorylation was noted in all the treated groups. Downregulation of the signal transduction pathways as ECM–receptor interactions and as TGF-beta WNT, JAK/STAT, cAMP and calcium signalling pathways was detected. Our results showed a downregulation of apoptosis, cellular senescence and focal adhesion pathways and in the pathways related to the immune system. On the other hand, the PPAR signalling pathway was upregulated in all of the animals after the treatments (Figure 1 and Figure 2).

### 3.2. Treatment Effects on Histology

**Figure 3:**
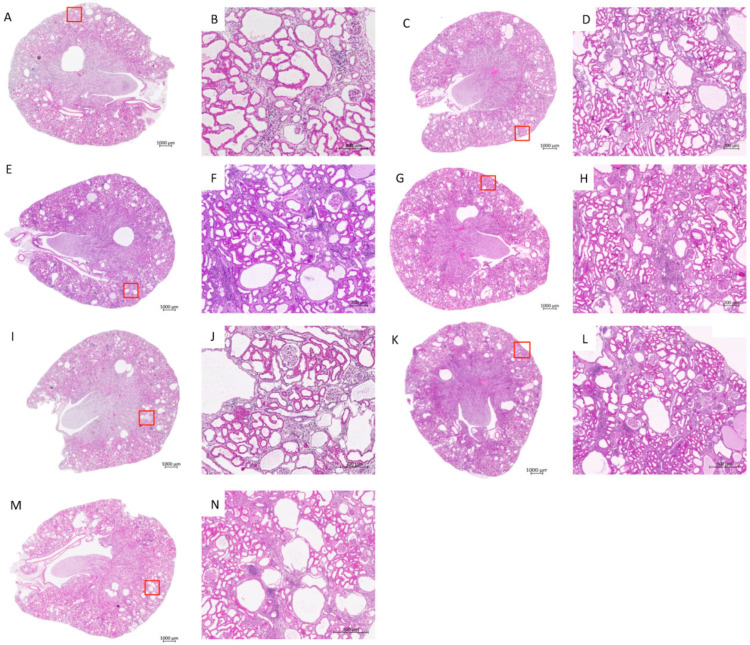
Haematoxylin and eosin (H&E) staining of PKD/Mhm (Cy/+) rat kidneys. Altered renal morphology in (**A**) PKD/Mhm (Cy/+) rat and (**B**) corresponding magnification of cyst details (red square); (**C**) ABCB5^+^-derived CoCM^+^ treated rat and (**D**) corresponding magnification of cyst details; (**E**) ASC-derived CM-treated rat and (**F**) corresponding magnification of cyst details; (**G**) i.p. ABCB5^+^-treated rat and (**H**) corresponding magnification of cyst details; (**I**) i.p. ASC-treated rat and (**J**) corresponding magnification of cyst details; (**K**) i.v. ABCB5^+^-treated rat and (**L**) corresponding magnification of cyst details; (**M**) i.v. ASC-treated rat and (**N**) corresponding magnification of cyst details. Images acquired with Axio Scan.Z1 microscope (ZEISS), 20× objective; (**B**,**D**,**F**,**H**,**J**,**L**,**N**) are zoomed in images of the area used to check the size of the scale bar.

**Figure 4:**
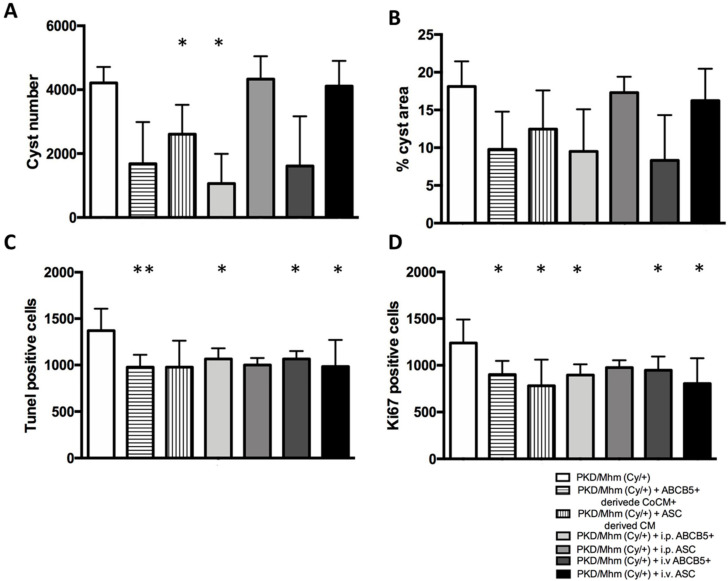
Histological changes in PKD/Mhm (Cy/+) rats after treatments (n = 6 in each group). Changes in cyst number (**A**), percentage (%) of cyst area (**B**), Tunel-positive cells (**C**) and Ki67-positive cells (**D**). Histograms show means ± Std.Dev. * p < 0.05, ** p < 0.005.

Despite no change in the kidney weight being noticed among the different groups (data not shown), H&E staining showed a decrease in cyst numbers in all of the treated groups, with the exception of intraperitoneal (i.p.) ASC and or intravenous (i.v.) ASC groups. In particular, cyst number was decreased by 1.6-fold and 3.9-fold in the ASC-derived CM and i.p. ABCB5^+^ groups, respectively (Figure 3 and Figure 4A), and the percentage of renal tissue covered by cyst was proportionally reduced in the same treated animals (Figure 4B).

Our results outlined a significant reduction in apoptotic positive cells in the kidney section in almost all treated groups except the ASC-derived CM and i.p. ASC groups (Figure 4C) and a significant reduction in proliferative positive cells, with the exception of the i.p. ASC group (Figure 4D).

### 3.3. Treatment Effects on Glomerular Function

To monitor the effect of the treatments and the health of the animals, we performed monthly plasma and urine biochemistry analyses and a transcutaneous GFR measurement.

#### 3.3.1. Plasma Biochemistry

**Figure 5:**
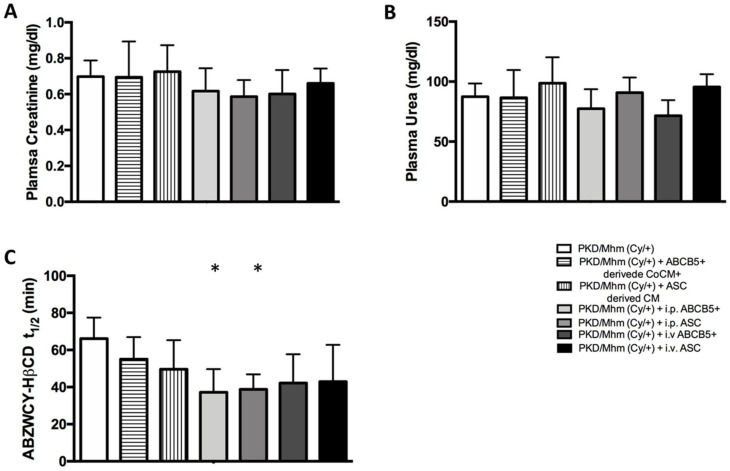
Effect of different treatments on renal function parameters in the PKD/Mhm (Cy/+) model (n = 6 in each group). Changes in plasma creatinine (**A**) and urea (**B**) concentrations, and in ABZWCY-HβCD half-life (t_1/2_) (**C**). Histograms show means ± Std.Dev. * p < 0.05.

In the evaluation of the renal function, plasma creatinine and urea are considered standard biomarkers. Our results showed a reduction in creatinine levels by 1.2-fold in the ABCB5^+^-derived CoCM^+^, i.p. ABCB5^+^, i.v. ABCB5^+^ and i.p. ASC groups at day 53 when compared with the untreated groups (Table S1), while on day, 167 we noted a slight decrease only in the i.p. ABCB5^+^ and i.p. ASC groups (Figure 5A). A slight reduction in urea levels was registered in the ABCB5^+^-derived CoCM^+^, i.p. ABCB5^+^ and i.v. ABCB5^+^ groups when compared with the untreated group (Table S1). The urea levels in the i.p. ABCB5^+^ and i.v. ABCB5^+^ groups remained lower until the end of the experiments (day 167). Conversely, conflicting results were observed in the ASC-derived CM, i.p. ASC and i.v. ASC groups, where these levels increased (Figure 5B). A decline in cholesterol levels was detected at day 109 in all the treated groups when compared with the untreated groups, notably a significant reduction in the i.p. ABCB5^+^ and i.v. ABCB5^+^ groups (*p* < 0.05) (Table S1). On day 167, the cholesterol levels of the treated groups were reduced in comparison with the untreated group, except for the ASC-derived CM group, which showed no difference. At the end of the experiment, we also noted a slight reduction in the triglycerides levels in all the treated animals (Table S1). The effects of the treatments were also noted in the plasma electrolytes. The sodium and calcium levels visibly decreased in the i.p. ABCB5^+^ group on day 167 (*p* < 0.05). At the same time point, a reduction in the sodium levels was observed also in the i.v. ABCB5^+^ group (*p* < 0.05) (Table S1).

#### 3.3.2. Transcutaneous GFR Measurement

The ABZWCY-HβCD elimination curve (t_1/2_) was used to assess the kidney function. On day 109, ABZWCY-HβCD t_1/2_ was reduced by 1.2-fold in the ASC-derived CM and i.v. ASC groups and by 1.5-fold in the i.p. ASC., i.p. ABCB5^+^ and i.v. ABCB5^+^ groups when compared with the untreated group (Table S2). On day 167, we noted a significant reduction in ABZWCY-HβCD t_1/2_ in the i.p. ABCB5^+^ and i.p. ASC groups (*p* < 0.05) (Figure 5C), indicating an improved GFR value.

#### 3.3.3. Urine Biochemistry

**Figure 6:**
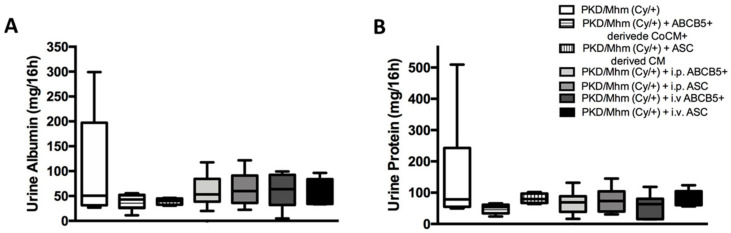
Effect of different treatments on urine parameters in the PKD/Mhm (Cy/+) model (n = 6 in each group). Changes in urine albumin (**A**) and protein (**B**) concentrations. Histograms show means ± Std.Dev.

The PKD/Mhm (Cy/+) rats are characterized by high levels of proteinuria and albuminuria. Our results showed a reduction in these parameters in all of the treated groups on day 167 when compared with the untreated group (Figure 6). Already, on day 109, proteinuria was reduced by at least 1.3-fold in the i.p. ABCB5^+^, i.v. ABCB5^+^ and ABCB5^+^-derived CoCM^+^ groups. A slight decrease was also detected at the same time point in the ASC-derived CM and i.p. ASC groups. A reduction of 1.4-fold of albuminuria was noticed in all of the treated groups on day 81. The levels rose on days 109 and 137 and decreased again at the end of the experiment (Table S3).

## 4. Discussion

CKD represents a group of chronic disorders characterized by some common pathological features. Among all of the CKD, the most prevalent and clinically significant are the ADPKD, ARPKD and NPHP [1]. Annually, the rate of patients with CKD increases by 7%, making CKD a worldwide public health problem [39]. Due to the poor availability of efficient therapies, affected patients can only undergo dialysis or kidney transplantation. For this reason, CKD has a huge human and economic impact on society and medical community [39,40]. A promising therapeutic approach is represented by stem cell applications, extensively used in recent years as a possible therapy for other kidney disorders [26]. Human MSCs are known for their immunomodulatory capability, which allows them to escape from the host’s immune system, facilitating inter-species transplantation. Given their capacity and the potential clinical application, testing human MSC in animals appears to be a logical way to proceed.

Over the years, the PKD/Mhm (Cy/+) model has been widely used to understand and characterize the progression and development of ADPKD and NPHP16 and to test new medical targets [18,41,42]. In this study, we tested the potential therapeutic effects of human stromal cells and their derived conditioned media in PKD/Mhm (Cy/+) rats. We repetitively administrated the cells either by i.v. or i.p. injection. The i.v. injection is the most common route of administration and considered by many to be a less invasive procedure. However, with the i.v. injection cells might be trapped in the lungs with the risk of causing emboli [33,43,44,45,46]. This is the reason why we also tested the i.p. injection. Overall, it appears to be an efficient and safe alternative route of administration. Moreover, in this study, we investigated the histological changes and analysed the renal function parameters.

A gene expression analysis was performed to examine the effects of ABCB5^+^, ASC, ABCB5^+^-derived CoCM^+^ and ASC-derived CM on the kidney. Several studies highlighted a reprogramming of metabolism in the PKD/Mhm (Cy/+) model, enhancing the aerobic glycolysis pathways rather than the oxidative phosphorylation [47]. This behaviour, which is typical of tumour cells, is known as the Warburg effect, and usually, it is correlated with a defect in the mitochondrial activity [47,48,49,50,51]. It is known that mitochondria are able to produce energy thanks to the oxidative phosphorylation pathway. Even if the dysfunctional activity of mitochondria is a well-described phenomenon in CKD [52,53,54,55], it was not possible to test it in this study. Nevertheless, we noted profound changes in pathways related to the metabolism in all of the experimental groups. Oxidative phosphorylation, citrate cycle, and the gluconeogenesis pathways and the pyruvate metabolism pathway were upregulated. Moreover, several studies demonstrated the correlation between the downregulation of the oxidative phosphorylation pathway and the reduction in the activity of PPARα and its co-activator, which are involved in the modulation of the energy metabolism [49,56,57,58]. Our results showed an upregulation of the PPAR signalling pathway, compatible with the upregulation of the oxidative phosphorylation pathway.

Besides metabolic reprogramming, the PKD/Mhm (Cy/+) animal model is also characterized by an incorrect response of the injury repair and inflammation mechanisms. In 2007, Weimbs suggested that a reduction in the *Polycystin 1* gene might be able to activate cell proliferation, leading to cyst formation [59]. This and the resultant fluid secretion and size increase could drive an abnormal production of growth factors, an alteration in cellular proliferation, the deposition of fibrotic tissue and the dedifferentiation of renal epithelial cells [59,60,61]. An important role in the cyst formation is played by the PI3K-Akt signalling pathway, usually upregulated, which promotes cellular proliferation and regulates the polarization and tissue expansion [2,62,63,64,65]. RNAseq results showed a downregulation of the pathway in all of the treated groups. Additionally, our results showed a downregulation of the TGF-β and WNT signalling pathways, and a downregulation of apoptosis. While the TGF-β pathway promotes cellular growth and differentiation, the WNT pathway regulates cellular proliferation, polarity and migration. The downregulation of all these pathways leads to the assumption that a restoration of the physiological cellular response has been triggered. As aforementioned, the inflammation mechanisms correspond to cyst formation and growth. In both the human ADPKD and PKD/Mhm (Cy/+) rat models, the NF-ĸB, JAK-STAT and TNF pathways are the major inflammatory pathways upregulated [66,67,68,69]. NF-ĸB is a promising target for treatment. In fact, in order to slow the disease progression, several studies tried to inhibit the NF-ĸB pathways through anti-inflammatory compounds [70,71]. In this study, we noted a downregulation of NF-ĸB, JAK-STAT and TNF after treatment. Moreover, we noted a downregulation of immune system-related pathways in the i.v. and i.p. ABCB5^+^ groups along with the ABCB5^+^-derived CoCM^+^ groups. A comparable pattern has also been noted in the i.p. and i.v. ASC groups. All together, these results suggest an attenuation of the inflammation. The loss of *Polycystin* gene function also causes an imbalance in calcium homeostasis in the cells, which may activate an abnormal cAMP response [72]. Despite no genetic therapy on specific genes being performed in this study, the restoration of the principal metabolic-related pathways also led to a downregulation of the cAMP and calcium signalling pathways. It is also known that an aberrant increase in cAMP levels might trigger activation of the RAS signalling pathway in the renal epithelial cells [2]. In line with the downregulation of the cAMP and calcium signalling pathways, our results also showed a downregulation of the RAS signalling pathway in all of the treated groups.

Based on the literature [55], the amelioration of both pathways involved in the metabolism and the downregulation of the cAMP and calcium signalling pathways may be correlated with a reduction in the cyst growth and number. To validate this hypothesis, we performed a morphological evaluation of whole kidney sections stained with H&E. Our results highlighted a decrease in the number of cysts in all of the treated groups, with the exception of the i.p. ASC group and a proportionate reduction in the cyst percentage area on the tissue.

Moreover, the concentration of calcium in the plasma was reduced in the ABCB5^+^-derived CoCM^+^ group and in both the i.p. and i.v. ABCB^+^ and i.p. and i.v. ASC groups. Keeping the aforementioned discussion in view, we were unimpressed to find out that the treated groups showing downregulation of the calcium and cAMP signalling pathways also exhibit reduced cyst numbers and lower plasma concentrations of calcium.

An alteration of the cellular cycle, resulting in increasing cell proliferation and apoptosis, is also well documented in the PKD/Mhm (Cy/+) model [73,74]. Based on this result and on our RNAseq results, we performed a co-staining of Ki67 and Tunel on kidney sections embedded in paraffin in order to evaluate the potential changes after the treatments. We noted a significant reduction in apoptotic and proliferative maker positive cells in all of the treated groups.

During the whole experiment, biochemistry analyses and transcutaneous GFR measurements were performed to follow up on the kidney function and more in general the health of the animals. Plasma biochemistry analyses showed a minor reduction in creatinine levels already on day 53, which remained so in the i.p. ABCB5^+^ and i.p. ASC groups until day 167. We also noted an improvement in the plasma levels of cholesterol and triglycerides.

However, since several elements (such as age, gender, hepatic function and muscle mass) may influence the plasma creatinine levels [75,76], we also monitored kidney function using an innovative and non-invasive method: the transcutaneous GFR measurement. At the end of the experiments, we noted an improvement in the kidney function. In fact, ABZWCY-HβCD half-life was reduced by 1.5-fold in both the i.v. ABCB5^+^ and ASC groups, by 1.6-fold in the i.p. ABCB5^+^ group, and by 1.7-fold in the i.p. ASC group. A slight amelioration was also detected in the ABCB5^+^-derived CoCM^+^ and ASC-derived CM groups. Additionally, small changes in albuminuria and proteinuria were detected in all of the treated groups.

To conclude, our study provides a wide description of the gene profile outcomes obtained by treating PKD/Mhm (Cy/+) rats with ABCB5^+^ or ASC cells and derived conditioned media, suggesting that ABCB5^+^ or ASC cell administration, and ABCB5^+^-derived CoCM^+^ and ASC-derived CM treatments might be efficient alternative therapies for cystic kidney disease. Numerous studies have suggested that the first mode of action of MSC is the secretion of paracrine factors to enhance tissue regeneration [77,78]. ASC and ABCB5^+^ are characterized by immunomodulatory and immunosuppressive activities, through the secretion of immunosuppressive cytokines [34,79,80,81]. Recently, the potential therapeutic role of factors released by the cells in these media has been investigated. Proteomic analyses highlighted the presence of chemokines, cytokines, angiogenic and growth factors in the MSC-conditioned media. For this reason, we also investigated the effects of ABCB5^+^-derived CoCM^+^ and ASC-derived CM. These healing properties of the cells and the factors that they released in the media led to an improvement in the inflammatory state in the animals. Moreover, secreted factors such a cytokines may have helped in the reprogramming of metabolism-related pathways and the reduction in cyst size. It is known, in fact, that a downregulation of metabolism pathways is related to cyst formation and development [69]. Furthermore, we demonstrated that i.p. administration is a more efficient alternative route than i.v. RNAseq technology in gene expression analyses and allowed us to be the first to obtain the most complete overview into possible mechanisms of stem cell therapy in PKD/Mhm (Cy/+) animals. Nevertheless, further studies have to be performed to test the proper dose of administration and to better understand the mode of action of the ABCB5^+^ and ASC cells, and their derived conditioned media. Future studies are needed to investigate their mitochondrial activity. No significant changes in plasma creatinine and urea were observed after the treatments, but the amelioration of kidney function is a very encouraging result.

The use of stem cell treatments in CKD and PKD can provide the basis for new therapeutic approach to these pathologies.

## Figures and Tables

**Table 1:** GSEA pathway analyses showing the numbers of up- and downregulated pathways in PKD/Mhm (Cy/+) groups (n = 6 in each group). (A) ABCB5^+^-derived CoCM^+^; i.p. ABCB5^+^; i.v. ABCB5^+^; (B) ASC-derived CM; i.p. ASC; i.v. ASC. Total analysed pathways are 304 in the ABCB5^+^-derived CoCM^+^; i.p. ABCB5^+^; i.v. ABCB5^+^ groups while 305 in the ASC-derived CM; i.p. ASC; i.v. ASC groups because GSEA analyses were performed in two different years.

**ABCB5^+^ Animal Group**		
	Total analysed pathways	304
+ABCB5^+^-derived CoCM^+^	Significantly regulated pathways (*adj*. *p*-value < 0.05)	185
	Significantly upregulated pathways (*adj. p*-value < 0.05)	37
	Significantly downregulated pathways (*adj. p*-value < 0.05)	148
+i.p. ABCB5^+^	Significantly regulated pathways (*adj. p*-value < 0.05)	165
	Significantly upregulated pathways (*adj. p*-value < 0.05)	46
	Significantly downregulated pathways (*adj. p*-value < 0.05)	119
+i.v. ABCB5^+^	Significantly regulated pathways (*adj. p*-value < 0.05)	142
	Significantly upregulated pathways (*adj. p*-value < 0.05)	47
	Significantly downregulated pathways (*adj. p*-value < 0.05)	95
**ASC Animal Group**		
	Total analysed pathways	305
+ASC-derived CM	Significantly regulated pathways (*adj. p*-value < 0.05)	42
	Significantly upregulated pathways (*adj. p*-value < 0.05)	31
	Significantly downregulated pathways (*adj. p*-value < 0.05)	11
+i.p. ASC	Significantly regulated pathways (*adj. p*-value < 0.05)	101
	Significantly upregulated pathways (*adj. p*-value < 0.05)	55
	Significantly downregulated pathways (*adj. p*-value < 0.05)	46
+i.v. ASC	Significantly regulated pathways (*adj. p*-value < 0.05)	122
	Significantly upregulated pathways (*adj. p*-value < 0.05)	48
	Significantly downregulated pathways (*adj. p*-value < 0.05)	74

## Data Availability

The gene expression data were submitted to the Gene Expression Omnibus with the following accession number: GSE161093.

## Supplementary Materials

The following supporting information can be downloaded at https://www.mdpi.com/article/10.3390/jcm11092601/s1. Table S1. Changes in plasma biochemistry in PKD/Mhm (Cy/+) groups (n = 6 in each group); Table S2. Changes in the ABZWCY-HβCD half-life in PKD/Mhm (Cy/+) groups (n = 6); Table S3. Changes in the urine protein and albumin concentrations in PKD/Mhm (Cy/+) groups (n = 6 in each group).
