# Supplementary material for: Potential Therapeutic Effects of Long-Term Stem Cell Administration: Impact on the Gene Profile and Kidney Function of PKD/Mhm (Cy/+) Rats
Source: J Clin Med. 2022 May 5;11(9):2601. doi: 10.3390/jcm11092601 (PMC9102853; doi:10.3390/jcm11092601)
Supplement: Supplementary file 1 [file jcm-11-02601-s001.zip › jcm-1699975-supplementary.pdf]

Table S1. Changes in plasma biochemistry in PKD/Mhm (Cy/+) groups (n=6 in each group).

Comparison between A) ABCB5^{+} derived CoCM^{+}; i.p. ABCB5^{+}; i.v. ABCB5^{+} and untreated groups; B) ASC derived CM; i.p. ASC; i.v. ASC and untreated groups.

Data are shown as mean ± Std.Dev. Values significantly different from control are indicated as *$p<0.05$, **$p<0.005$.

A	Parameter	Animal group	Baseline	Day25	Day 53	Day 81	Day 109	Day 137	Day 167
	Creatinine	Untreated	0.3 ± 0.1	0.4 ± 0.1	0.6 ± 0 1	0.6 ± 0.2	0.6 ± 0.1	0.7 ± 0.1	0.7 ± 0.1
	(mg/dl)	+ABCB5^{+} derived CoCM^{+}	0.3 ± 0.1	0.5 ± 0.2	0.5 ± 0.1	0.5 ± 0.1	0.7 ± 0.2	0.6 ± 0.1	0.7 ± 0.2
		+ i.p. ABCB5^{+}	0.3 ± 0.1	0.5 ± 0.1	0.5 ± 0.1	0.5 ± 0.1	0.6 ± 0.1	0.6 ± 0.1	0.6 ±0.1
		+ i.v. ABCB5^{+}	0.3 ± 0.1	0.4 ± 0.1	0.4 ± 0.1	0.5 ± 0.2	0.5 ± 0.1	0.5 ± 0.1	0.7 ± 0.1
	Urea	Untreated	68.4 ± 18.2	69.4 ± 9.9	73.9 ± 8.6	88.4 ± 11.7	85.6 ± 8.1	85.1 ±13.0	87.4 ± 11.0
	(mg/dl)	+ABCB5^{+} derived CoCM^{+}	57.5 ± 17.6	68.2 ± 19.9	80.5 ± 15.6	73.9 ± 20.8	74.8 ± 17.2	83.7 ± 20.9	86.5 ± 23.2
		+ i.p. ABCB5^{+}	56.1 ± 10.4	66.6 ± 19.2	67.2 ± 14.7	68.9 ± 17.7	75.3 ± 16.0	74.9 ± 17.0	79.6 ± 17.7
		+ i.v. ABCB5^{+}	52.2 ± 16.2	55.3 ± 17.6	59.3 ± 15.6	65.0 ± 20.2	63.9 ± 18.6	66.8 ± 23.6	72.3 ± 12.4
	Na	Untreated	144.3 ± 0.8	143.0 ± 3.6	142.3 ±6.9	141.3 ± 2.3	144.3 ± 2.3	141.8 ± 1.7	144.8 ± 1.9
	(mmol/l)	+ABCB5^{+} derived CoCM^{+}	141.2 ± 2.9	144.3 ± 3.6	144.0 ± 2.8	142.5 ± 3.1	144.3 ± 1.0	142.3 ± 3.2	142.2 ± 1.7
		+ i.p. ABCB5^{+}	143.3 ± 1.6	142.7 ± 2.3	144.3 ± 2.4	143.3 ± 3.2	144.3 ± 1.4	143.7 ± 2.5	**141.9 ± 1.6***
		+ i.v. ABCB5^{+}	141.6 ± 2.2	142.7 ± 2.0	143.7 ± 2.8	143.1 ± 2.3	143.7 ± 1.3	143.0 ± 2.6	**142.0 ± 2.0***
	K	Untreated	5.4 ± 0.3	5.1 ± 0.6	5.2 ± 0.7	5.3 ± 0.4	5.3 ± 0.2	5.7 ± 0.4	5.1 ± 0.2
	(mmol/l)	+ABCB5^{+} derived CoCM^{+}	5.2 ± 0.2	5.0 ± 0.2	5.0 ± 0.3	5.1 ± 0.4	4.9 ± 0.3	5.2 ± 0.3	5.3 ± 0.4
		+ i.p. ABCB5^{+}	5.3 ± 0.3	4.8 ± 0.1	5.0 ± 0.2	5.3 ± 0.6	**5.2 ± 0.2***	**5.1 ± 0.3***	4.9 ± 0.2
		+ i.v. ABCB5^{+}	5.2 ± 0.2	4.8 ± 0.2	4.9 ± .0.2	5.2 ± 0.3	4.9 ± 0.3	**5.1 ± 0.3***	5.0 ± 0.2
	Ca	Untreated	2.5 ± 0.2	2.6 ± 0.0	2.6 ± 0.1	2.6 ± 0.1	2.6 ± 0.0	2.6 ± 0.1	2.7 ± 0.1
	(mmol/l)	+ABCB5^{+} derived CoCM^{+}	2.6 ± 0.1	2.6 ± 0.1	2.5 ± 0.1	5.5 ± 7.3	**2.6 ± 0.0***	2.6 ± 0.1	2.4 ± 0.2
		+ i.p. ABCB5^{+}	2.5 ± 0.1	**2.5 ± 0.1***	2.5 ± 0.2	2.5 ± 0.2	**2.5 ± 0.1****	2.7 ± 0.0	**2.4 ± 0.2***
		+ i.v. ABCB5^{+}	2.5 ± 0.2	2.5 ± 0.1	2.5 ± 0.2	2.4 ± 0.3	2.6 ± 0.1	2.7 ± 0.5	2.5 ± 0.2
	PO_4	Untreated	2.4 ± 0.5	2.2 ± 0.1	2.6 ± 0.2	2.1 ± 0.6	2.0 ± 0.3	2.1 ± 0.2	2.1 ± 0.3
	(mmol/l)	+ABCB5^{+} derived CoCM^{+}	2.6 ± 0.3	2.5 ± 0.1	2.4 ± 0.2	2.3 ± 0.2	2.1 ± 0.2	2.1 ± 0.2	2.2 ± 0.2
		+ i.p. ABCB5^{+}	2.7 ± 0.2	2.5 ± 0.1	**2.5 ± 0.1***	2.1 ± 0.2	2.1 ± 0.2	2.1 ± 0.1	2.1 ± 0.1
		+ i.v. ABCB5^{+}	2.5 ± 0.3	2.4 ± 0.2	2.3 ± 0.1	2.2 ± 0.2	2.1 ± 0.2	2.0 ± 0.1	1.9 ± 0.2
	Cholesterol	Untreated	97.3 ± 7.5	99.7 ± 6.7	110.0 ± 9.1	107.5 ± 8.5	131.7 ± 10.0	131.7 ± 10.2	154.3 ± 15.5
	(mg/dl)	+ABCB5^{+} derived CoCM^{+}	89.7 ± 5.9	117.4 ± 39.0	102.3 ± 9.7	102.7 ± 18.6	110.5 ± 22.3	121.5 ± 16.7	138.3 ± 30.9
		+ i.p. ABCB5^{+}	88.3 ± 4.6	101.3 ± 25.8	102.7 ± 12.5	97.7 ± 10.9	**100.3 ± 12.6***	116.0 ± 14.2	135.1 ± 23.0
		+ i.v. ABCB5^{+}	88.6 ± 6.9	105.9 ± 37.4	94.0 ± 14.6	100.9 ± 22.7	**104.4 ± 17.0***	109.9 ± 18.7	134.0 ± 29.3
	Triglycerides	Untreated	69.2 ± 26.3	54.3 ± 14.5	67.2 ± 19.4	91.5 ± 21.6	68.7 ± 10.0	60.2 ± 15.0	108.8 ± 48.3
	(mg/dl)	+ABCB5^{+} derived CoCM^{+}	75.7 ± 34.5	49.8 ± 19.2	44.3 ± 16.0	53.3 ± 24.6	60.7 ± 22.9	71.7 ± 32.0	69.8 ± 23.4
		+ i.p. ABCB5^{+}	77.7 ± 29.8	37.1 ± 7.8	49.0 ± 14.4	53.9 ± 23.3	72. 7 ± 42.7	69.0 ± 28.0	79.3 ± 29.0

	+ i.v. $ABCB5^{+}$	70.9 ± 30.7	52.0 ± 5.6	50.0 ± 13.2	56.9 ± 20.0	72.7 ± 34.3	72.9 ± 30.4	72.1 ± 29.6

B Parameter	Animal group	Baseline	Day25	Day 53	Day 81	Day 109	Day 137	Day 167
Creatinine	Untreated	0.3 ± 0.1	0.4 ± 0.1	0.6 ± 0 1	0.6 ± 0.2	0.6 ± 0.1	0.7 ± 0.1	0.7 ± 0.1
(mg/dl)	+ ASC derived CM	0.3 ± 0.1	0.5 ± 0.1	0.7 ± 0.1	0.7 ± 0.1	0.7 ± 0.0	0.7 ± 0.1	0.7 ± 0.2
	+ i.p. ASC	0.3 ± 0.0	0.5 ± 0.1	0.5 ± 0.1	0.5 ± 0.1	0.5 ± 0.1*	0.6 ± 0.1	0.6 ± 0.1
	+ i.v. ASC	0.3 ± 0.0	0.5 ± 0.1	0.6 ± 0.0	0.7 ± 0.1	0.6 ± 0.0	0.7 ± 0.1	0.7 ± 0.1
Urea	Untreated	68.4 ± 18.2	69.4 ± 9.9	73.9 ± 8.6	88.4 ± 11.7	85.6 ± 8.1	85.1 ±13.0	87.4 ± 11.0
(mg/dl)	+ ASC derived CM	67.9 ± 8.2	11.8 ± 13.4	93.3 ± 7.3	97.6 ± 7.8	94.4 ± 7.0	96.4 ± 8.2	98.7 ± 21.6
	+ i.p. ASC	66.0 ± 8.9	74.7 ± 10.7	84.5 ± 7.8	80.8 ± 11.7	81.1 ± 11.5	79.2 ± 9.7	90.8 ± 12.7
	+ i.v. ASC	63.9 ± 3.6	80.1 ± 7.0	91.3 ± 7.4	88.9 ± 12.3	86.8 ± 6.2	90.9 ± 6.5	95.6 ± 10.6
Na	Untreated	144.3 ± 0.8	143.0 ± 3.6	142.3 ±6.9	141.3 ± 2.3	144.3 ± 2.3	141.8 ± 1.7	144.8 ± 1.9
(mmol/l)	+ ASC derived CM	144.8 ± 4.0	140.0 ± 3.6	138.6 ± 2.6	140.5 ± 2.5	143.0 ± 2.9	145.0 ± 2.7	145.3 ± 2.2
	+ i.p. ASC	143.2 ± 2.1	141.4 ± 4.9	139.8 ± 3.3	145.3 ± 5.1	144.7 ± 1.9	144.3 ± 0.8	**138.5 ± 5.0***
	+ i.v. ASC	144.0 ± 2.1	144.7 ± 3.5	140.0 ± 3.7	144.0 ± 6.5	143.8 ± 2.8	143.0 ± 0.9	139.5 ± 4.4
K	Untreated	5.4 ± 0.2	5.1 ± 0.6	5.2 ± 0.7	5.3 ± 0.4	5.3 ± 0.2	5.7 ± 0.4	5.1 ± 0.2
(mmol/l)	+ ASC derived CM	6.0 ± 1.8	5.2 ± 0.5	5.2 ± 0.3	5.4 ± 0.4	5.3 ± 0.5	5.2 ± 0.4	5.7 ± 0.4
	+ i.p. ASC	5.0 ± 0.3	5.4 ± 0.9	5.4 ± 0.3	5.3 ± 0.4	5.2 ± 0.2	5.2 ± 0.3	**6.1 ± 0.5****
	+ i.v. ASC	5.0 ± 0.4	5.9 ± 0.6	5.3 ± 0.5	5.2 ± 0.3	5.2 ± 0.4	5.0 ± 0.5	5.7 ± 0.4
Ca	Untreated	2.5 ± 0.2	2.6 ± 0.0	2.6 ± 0.1	2.6 ± 0.1	2.6 ± 0.0	2.6 ± 0.1	2.9 ± 0.1
(mmol/l)	+ ASC derived CM	1.5 ± 0.2	2.6 ± 0.1	2.6 ± 0.0	2.5 ± 0.1	2.6 ± 0.1	2.6 ± 0.1	2.7 ± 0.1
	+ i.p. ASC	2.6 ± 0.1	2.6 ± 0.1	2.6 ± 0.1	2.6 ± 0.1	2.6 ± 0.1	2.6 ± 0.1	2.6 ± 0.1
	+ i.v. ASC	2.7 ± 0.1	2.6 ± 0.1	2.5 ± 0.1	2.6 ± 0.0	2.6 ± 0.1	2.6 ± 0.1	2.6 ± 0.1
PO_4	Untreated	2.4 ± 0.5	2.2 ± 0.1	2.6 ± 0.2	2.1 ± 0.4	2.0 ± 0.3	2.1 ± 0.2	2.1 ± 0.3
(mmol/l)	+ ASC derived CM	2.5 ± 0.3	2.4 ± 0.2	2.3 ± 0.1	2.3 ± 0.1	2.3 ± 0.1	2.2 ± 0.2	2.0 ± 0.4
	+ i.p. ASC	2.6 ± 0.3	2.7 ± 0.3	2.3 ± 0.3	2.2 ± 0.1	2.1 ± 0.1	2.2 ± 0.1	1.8 ± 0.3
	+ i.v. ASC	2.7 ± 0.2	2.5 ± 0.4	2.2 ± 0.3	2.3 ± 0.1	2.2 ± 0.1	2.3 ± 0.2	1.9 ± 0.2
Cholesterol	Untreated	97.3 ± 7.5	99.7 ± 6.7	110.0 ± 9.1	107.5 ± 8.5	131.7 ± 10.0	131.7 ± 10.2	154.3 ± 15.5
(mg/dl)	+ ASC derived CM	99.8 ± 7.6	100.3 ± 9.7	107.4 ± 13.7	117.8 ± 12.8	127.0 ± 17.9	138.3 ± 23.4	152.3 ± 22.0
	+ i.p. ASC	95.8 ± 6.5	96.2 ± 7.8	100.5 ± 7.3	111.0 ± 5.4	111.0 ± 8.5	134.3 ± 5.7	144.7 ± 10.6
	+ i.v. ASC	97.0 ± 8.5	103.3 ± 5.1	99.7 ± 6.3	116.5 ± 8.4	119.0 ± 6.5	139.0 ± 13.1	149.5 ± 17.3
Triglycerides	Untreated	69.2 ± 26.3	54.3 ± 14.5	67.2 ± 19.4	91.5 ± 21.6	68.7 ± 10.0	60.2 ± 15.0	108.8 ± 48.3
(mg/dl)	+ ASC derived CM	78.5 ± 14.5	82.5 ± 27.0	62.8 ± 28.2	81.3 ± 31.3	66.0 ± 7.0	80.8 ± 21.9	63.3 ± 36.2
	+ i.p. ASC	76.5 ± 17.8	77.0 ± 22.8	64.5 ± 23.1	99.0 ± 43.2	72.0 ± 16.6	78.0 ± 14.2	98.8 ± 42.2
	+ i.v. ASC	94.7 ± 28.1	93.0 ± 16.6	70.3 ± 15.0	70.7 ± 15.0	10.2 ± 30.7	85.7 ± 27.0	63.5 ± 17.2

Table S2. Changes of ABZWCY-HβCD half-life in PKD/Mhm (Cy/+) groups (n=6).

Comparison between ABCB5+ derived CoCM+, i.v. or i.p. ABCB5+ groups and untreated. Data are shown as mean ± Std.Dev. Values significantly different from control are indicated as *$p<0.05$*.

A Parameter	Animal group	Baseline	Day25	Day 53	Day 81	Day 109	Day 137	Day 167
ABZWCY-HβCD	Untreated	26.7 ± 9.3	30.6 ± 7.1	39.3 ± 11.3	42.3 ± 6.4	51.4 ± 16.8	60.7 ± 29.9	66.0 ± 11.4
$t_{1/2}$ (min)	+ABCB5$^+$ derived CoCM$^+$	24.0 ± 7.6	24.9 ± 5.6	53.3 ± 28.5	47.2 ± 15.1	52.0 ± 22.7	47.1 ± 25.8	55.0 ± 12.0
	+ i.p. ABCB5$^+$	25.3 ± 9.8	31.7 ± 13.2	38.6 ± 10.7	43.6 ± 13.1	32.7 ± 8.6	39.0 ± 18.1	**41.2 ± 15.5***
	+ i.v. ABCB5$^+$	23.8 ± 4.4	27.0 ± 9.5	32.5 ± 17.0	45.0 ± 15.3	35.2 ± 13.8	38.8 ± 11.1	43.9 ± 19.1

B Parameter	Animal group	Baseline	Day25	Day 53	Day 81	Day 109	Day 137	Day 167
ABZWCY-HβCD	Untreated	26.7 ± 9.3	30.6 ± 7.1	39.3 ± 11.3	42.3 ± 6.4	51.4 ± 16.8	60.7 ± 29.9	66.0 ± 11.4
t1/2 (min)	+ ASC derived CM	26.8 ± 7.3	33.4 ± 11.9	38.5 ± 9.6	42.6 ± 9.1	43.0 ± 10.7	51.2 ± 16.8	49.6 ± 15.7
	+ i.p. ASC	34.6 ± 5.7	28.3 ± 5.2	31.1 ± 8.8	40.8 ± 8.2	36.5 ± 9.6	39.3 ± 10.8	**38.8 ± 8.1***
	+ i.v. ASC	33.2 ± 7.3	40.28 ± 12.2	31.1 ± 10.1	49.9 ± 23.3	43.0 ± 16.8	54.0 ± 11.2	43.0 ± 19.8

Table S3. Changes in urine protein and albumin concentration in PKD/Mhm (Cy/+) groups (n=6 in each group).

Comparison between A) ABCB5$^+$ derived CoCM$^+$; i.p. ABCB5$^+$; i.v. ABCB5$^+$ and untreated groups; B) ASC derived CM; i.p. ASC; i.v. ASC and untreated groups. Data are shown as mean ± Std.Dev. Values significantly different from control are indicated as *$p<0.05$*.

A Parameter	Animal group	Baseline	Day25	Day 53	Day 81	Day 109	Day 137	Day 167
Protein	Untreated	10.6 ± 5.2	19.5 ± 5.2	27.7 ± 9.8	32.7 ± 4.6	61.4 ± 16.3	96.8 ± 26.5	154.5 ± 177.7
(mg/16h)	+ABCB5$^+$ derived CoCM$^+$	12.1 ± 5.5	19.7 ± 6.8	25.4 ± 12.9	33.8 ± 12.2	41.9 ± 16.7	50.2 ± 20.6	49.8 ± 16.4
	+ i.p. ABCB5$^+$	12.2 ± 2.3	19.1 ± 5.2	30.2 ± 12.7	36.4 ± 13.1	47.5 ± 19.5	51.9 ± 22.6	68.4 ± 35.0

	+ i.v. ABCB5^{+}	13.0 ± 3.5	20.3 ± 6.9	26.1 ± 10.2	34.4 ± 17.3	36.9 ± 19.5	**39.4 ± 25.6***	58.8 ± 36.5
Albumin	Untreated	2.3 ± 1.5	4.3 ± 1.7	12.4 ± 4.1	22.4 ± 11.4	27.4 ± 13.2	25.4 ± 11.3	103.9 ± 108.5
(mg/16h)	+ABCB5^{+} derived CoCM^{+}	1.8 ± 1.1	4.5 ± 2.0	9.4 ± 4.5	15.9 ± 6.4	24.8 ± 13.1	32.5 ± 26.4	39.1 ± 16.2
	+ i.p. ABCB5^{+}	1.9 ± 0.9	8.6 ± 5.0	14.4 ± 12.1	21.4 ± 13.2	30.6 ± 24.7	38.9 ± 23.0	58.1 ± 37.5
	+ i.v. ABCB5^{+}	1.6 ± 1.5	5.5 ± 3.7	9.1 ± 6.6	14.3 ± 8.7	20.9 ± 15.1	26.5 ± 24.6	52.6 ± 37.7

B Parameter	Animal group	Baseline	Day25	Day 53	Day 81	Day 109	Day 137	Day 167
Protein	Untreated	10.6 ± 5.2	19.5 ± 5.2	27.7 ± 9.8	32.7 ± 4.6	61.4 ± 16.3	96.8 ± 26.5	154.5 ± 177.7
(mg/16h)	+ ASC derived CM	11.1 ± 3.2	18.6 ± 5.0	21.8 ± 12.0	35.6 ± 15.0	53.3 ± 18.3	63.7 ± 17.9	81.3 ± 15.8
	+ i.p. ASC	8.9 ± 3.2	18.8 ± 6.8	33.6 ± 5.6	38.6 ± 11.5	57.5 ± 15.7	66.2 ± 9.1	76.0 ± 41.1
	+ i.v. ASC	10.6 ± 5.3	14.9 ± 3.8	33.1 ± 13.5	36.2 ± 8.7	70.8 ± 32.0	88.1 ± 21.6	83.6 ± 25.5
Albumin	Untreated	2.3 ± 1.5	4.3 ± 1.8	12.4 ± 4.1	22.4 ± 11.4	27.4 ± 13.2	25.4 ± 11.3	103.9 ± 108.5
(mg/16h)	+ ASC derived CM	2.2 ± 1.9	6.6 ± 4.1	7.3 ± 4.8	16.0 ± 7.2	18.3 ± 16.3	30.2 ± 12.7	39.0 ± 6.6
	+ i.p. ASC	1.4 ± 0.9	4.7 ± 2.6	11.2 ± 6.2	18.0 ± 4.7	27.6 ± 17.2	31.0 ± 14.9	64.7 ± 34.6
	+ i.v. ASC	0.7 ± 0.2	3.5 ± 1.2	10.2 ± 5.4	11.6 ± 5.3	33.3 ± 15.3	28.6 ± 18.7	60.4 ± 26.1
